# Assessing COVID-19-Related Psychological Distress: Validation of the AMICO Scale in Spanish Nursing University Students

**DOI:** 10.3390/healthcare13091058

**Published:** 2025-05-04

**Authors:** Nadine Badillo-Sánchez, Cristina Morgado-Toscano, Regina Allande-Cussó, Juan Gómez-Salgado, Murat Yıldırım, Krzysztof Goniewicz, Israel Macías-Toronjo, Javier Fagundo-Rivera

**Affiliations:** 1 School of Doctorate, University of Huelva, 21007 Huelva, Spain; 2 Department of Nursing, University of Seville, 41009 Seville, Spain; 3 Department of Sociology, Social Work and Public Health, Faculty of Labour Sciences, University of Huelva, 21007 Huelva, Spain; 4 Safety and Health Postgraduate Programme, Universidad Espíritu Santo, Guayaquil 092301, Ecuador; 5 Department of Psychology, Faculty of Science and Letters, Ağrı İbrahim Çeçen University, 04100 Ağrı, Türkiye; 6 Psychology Research Center, Khazar University, 1009 Baku, Azerbaijan; 7 Department of Security, Polish Air Force University, 08-521 Deblin, Poland; 8 Department of Rehabilitation, FREMAP Huelva, 21001 Huelva, Spain; 9 Centro Universitario de Enfermería Cruz Roja, University of Seville, 41009 Seville, Spain

**Keywords:** COVID-19 pandemic, mental health, nursing students, psychological stress, emotional well-being, fear, anxiety, AMICO scale, validation study, assessment, health status, workplace stress, working and COVID-19

## Abstract

**Background**: The COVID-19 pandemic had a significant impact on nursing students by intertwining academic demands with health concerns, a situation that had effects on their emotional well-being and academic engagement. Factors such as sociodemographic characteristics and health status determined their experiences. Understanding these dynamics is crucial, especially in the context of the Spanish health and education systems. The present study aimed to adapt and assess the psychometric properties of the AMICO scale in the context of nursing students in Spain. **Methods**: Cross-sectional descriptive study. The study was carried out in University Nursing Centers in Spain using a non-probabilistic snowball sampling method. The total sample consisted of 1197 nursing students. Sociodemographic variables were included, as well as questions related to general health and some more specific questions about COVID-19. For the reliability study, Cronbach’s alpha was calculated. An exploratory factor analysis using principal components and varimax rotation was applied, excluding items with loadings below 0.05. **Results**: In this study, 1197 nursing students residing in Spain participated, of whom 85% were female, with a mean age of 22.35 years. Despite 73.9% of the students being isolated due to exposure to COVID-19, they rated their general health positively (7.86). Females reported higher levels of anxiety. The AMICO scale revealed significant differences according to gender, health, and vaccination history, showing high reliability (Cronbach’s alpha = 0.913). **Conclusions**: The two-factor structure of the AMICO scale was validated, confirming its suitability for assessing anxiety and fear among nursing students in Spain. The study revealed significant emotional distress during the COVID-19 pandemic, particularly among women, high-risk individuals, and those fully vaccinated. These findings accentuate the need for higher education institutions to implement targeted mental health interventions during public health emergencies. Future longitudinal research should examine the evolving psychological impact of such crises and the mitigating roles of quality of life, sleep, and physical activity.

## 1. Introduction

The COVID-19 pandemic profoundly transformed the educational landscape, with a particular impact on disciplines such as nursing, whose students are closely linked to healthcare environments [1,2]. The convergence of academic demands and health-related concerns significantly shaped students’ experiences during the crisis, affecting both their emotional well-being and academic engagement [3,4].

Academic engagement—defined as students’ active involvement, dedication, and sustained effort in their studies—was challenged by external stressors, which weakened motivation and focus [5,6,7,8]. Personal variables such as gender, age, place of residence, and household composition influenced the perceptions of safety and risk [9], while general health status and previous experiences with COVID-19 further conditioned emotional stability [10,11].

These factors overlapped with specific academic responsibilities [12], such as clinical placements, which imposed additional burdens on students in more advanced stages of their programs [13,14]. In these settings, perceived safety—shaped by protective measures and training in COVID-19 prevention—was critical to students’ willingness to engage in clinical practice [15,16].

The interplay of sociodemographic, health-related, and academic factors created a complex framework that influenced nursing students’ emotional responses, with fear and anxiety varying according to individual circumstances and available support systems [12,15,17,18]. Understanding this dynamic is essential for assessing the pandemic’s impact on students’ mental health and academic performance. Moreover, the effects of the COVID-19 pandemic on the educational sphere and academic placement settings were also observed in students involved in other university curricula, both within the healthcare sector [19] and in disciplines outside of it, such as teacher education [20].

Despite the growing body of international research, a specific gap remains in the literature regarding validated instruments for measuring COVID-19-related fear and anxiety in nursing students in Spain, a country with distinctive healthcare and educational structures, as well as varied regional responses to the pandemic [9,14,21,22].

Several psychometric instruments have been developed to assess mental health during the pandemic, including the Coronavirus Anxiety Scale (CAS) [23], the COVID-19 Stress Scale (CSS) [24], and the Fear of COVID-19 Scale (FCV-19S) [25], all of which have demonstrated utility in university settings [26,27,28]. However, the AMICO scale [29,30], a 16-item tool designed to assess both anxiety and fear related to COVID-19, has emerged as a particularly robust measure. The AMICO scale has a Cronbach’s alpha value of 0.92 and has been shown to be valid and reliable in various contexts, including specific subgroups such as the general adult population [30], pregnant women [31], and older adults [32] in Spain.

In this context, the present study aims to adapt and validate the AMICO scale for use among nursing students in Spain. A psychometrically sound instrument is essential to accurately measure the emotional impact of the pandemic on this group, providing a foundation for the development of targeted psychological support and preventive strategies within academic settings.

## 2. Materials and Methods

### 2.1. Design

A descriptive cross-sectional study was conducted for cultural adaptation and psychometric validation of the AMICO scale. The Herzog et al. classification [33] as well as the STROBE checklist were followed to design the study, report the assessment results, and revise the manuscript for publication [34].

### 2.2. Participants and Procedure

This study was carried out on Spanish nursing students. In Spain, a total of 1,333,567 university students were enrolled in the 2021–2022 academic year. Of these, 50,688 were enrolled in the Bachelor’s degree in Nursing [35].

The sample size was calculated on the basis of the population size, i.e., the number of undergraduate nursing students in Spain (50,688), with a confidence level of 95%, a precision of 3%, and an expected loss rate of 25%. The minimum sample size required to ensure statistical significance was 382 individuals; however, the final sample included 1197 nursing students from various provinces of Spain, 47 in total, including Alaba, Albacete, Alicante, Almeria, Asturias, Avila, Badajoz, Baleares, Barcelona, Burgos, Caceres, Cadiz, Cantabria, Castellon, Ceuta, Ciudad Real, Community of Madrid, Cordoba, Cuenca, Granada, Guadalajara, Guipuzcoa, and Huelva, among others. The inclusion criterion was being enrolled in a Bachelor’s degree in Nursing in Spain. Questionnaires that were not fully completed were excluded from the final sample.

Due to the specific characteristics of the sample and the necessary conditions for data collection, non-probabilistic snowball sampling was employed [36]. This method has certain disadvantages, including the lack of control over the sampling process, which may lead to sampling bias and limit the generalizability of the results to a broader population of nursing students [37]. However, it also offers advantages, such as reduced costs and shorter recruitment times when aiming to reach a sufficiently large population for studying a rare characteristic.

To recruit the sample, an online questionnaire was developed using the Google Forms© application. The questionnaire included information about the study, along with items related to the study variables. It was distributed to nursing students through a cover letter sent to the Deans of all Faculties offering a Bachelor’s degree in Nursing in Spain, via the National Conference of Deans of Nursing. It was also shared with the Spanish General Council of Nursing and the Spanish Association of Undergraduate Nursing Students, thereby reaching participants through official and authorized channels.

All subjects were duly informed about the purpose of the study, as well as about their voluntary, anonymous, and confidential participation, ensuring these conditions. The questionnaire was distributed between October 28, 2021, and May 31, 2022, reaching nursing students across 47 provinces of Spain.

### 2.3. Variables and Instruments

The questionnaire included sociodemographic variables (such as gender, age, province of residence, and number of cohabitants), general health questions, and more specific items related to COVID-19 (including contagion, isolation, and vaccination). Additionally, variables related to anxiety and fear of COVID-19, as well as academic factors (such as current academic year, type of university, and work experience), were incorporated. The inclusion of these variables was intended to capture the national diversity of nursing students across Spain, rather than to ensure representativeness of all Spanish universities. Although the sampling strategy and recruitment process did not allow for identifying the specific institutions in which participants were enrolled, data were collected on the type of academic institution (public, private, or affiliated), along with province and academic year. These variables served as proxies for institutional and geographic diversity, allowing for broad national coverage while maintaining participant anonymity and avoiding institutional-level identification.

The AMICO scale (Anxiety and Fear of COVID-19), originally developed during the third wave of the pandemic, was designed to evaluate psychological responses related to the COVID-19 crisis within the Spanish adult population [25,30]. This instrument, inspired by the FCV-19S scale, expanded its scope by explicitly incorporating anxiety symptoms linked to the pandemic. Its initial validation included a representative sample of 1036 adult participants residing in Spain. The final version of the scale comprises 16 items grouped into two dimensions—fear and anxiety—that together accounted for 64% of the total variance (KMO = 0.94; Bartlett’s test *p* < 0.001). Each item is rated on a 10-point Likert scale, from 1 (lowest perceived intensity) to 10 (highest perceived intensity), with the total score calculated as the average score of all item responses. Interpretation thresholds classify the results into low (≤ 4.31), moderate (4.32–6.4), and high (≥ 6.41) levels of anxiety and fear. The psychometric evaluation confirmed high internal consistency, with a Cronbach’s alpha of 0.92. Furthermore, in a subsequent application of the scale among Spanish nursing professionals, it demonstrated strong diagnostic performance, with a sensitivity of 90.48% and a specificity of 76% [38].

### 2.4. Data Analysis

Descriptive analyses, both univariate and bivariate, were conducted using SPSS Statistics © v26 software [39]. Since the Kolmogorov–Smirnov test yielded a p-value below 0.05, the data were considered to deviate significantly from a normal distribution. Accordingly, non-parametric methods were employed for hypothesis testing, specifically the Mann–Whitney U and Kruskal–Wallis tests, while Kendall’s Tau-b coefficient was used to assess associations between pairs of quantitative variables.

The decision to use non-parametric tests was not based solely on the rejection of normality. Although the sample size was large (*n* = 1197), the AMICO scale scores originated from a Likert-type response format, which, despite being treated as continuous in some psychometric contexts, inherently reflects an ordinal structure. This challenges key assumptions of parametric testing, such as interval-level measurement and homoscedasticity. Moreover, in addition to the statistical test, visual inspection of the data confirmed a skewed distribution. Therefore, the use of non-parametric methods was deemed both cautious and methodologically appropriate for the characteristics of the data.

To examine the factorial structure of the AMICO scale, an exploratory factor analysis (EFA) was performed using principal axis factoring, a method recommended when data deviate from normality and a stable factor solution is required [40]. Promax rotation was applied to allow for correlation between the extracted factors, and the final factor structure was determined by selecting the items with the highest loadings within each dimension. The number of factors retained was based on the Kaiser–Guttman criterion, considering only those with eigenvalues greater than 0.924. Subsequently, a confirmatory factor analysis (CFA) was conducted to validate the structure, applying the most widely accepted fit indices, including chi-squared/df (χ^2^/df < 3.0), the Comparative Fit Index (CFI ≥ 0.90), the Tucker–Lewis Index (TLI ≥ 0.90), also referred to as the Non-Normal Fit Index (NNFI ≥ 0.90), the Incremental Fit Index (IFI ≥ 0.90), the Root-Mean-Square Error of Approximation (RMSEA ≤ 0.08), and the Standardized Root-Mean-Square Residual (SRMR ≤ 0.08) [41].

To evaluate the internal consistency of the scale, Cronbach’s alpha was initially calculated. Additionally, following recent methodological recommendations for reliability assessment, McDonald’s omega coefficient was computed, as it provides a more robust estimation under the assumption of tau-equivalence. This coefficient was also adjusted to account for the impact of correlated errors on the reliability indices [42]. Furthermore, inter-item correlations were calculated to assess the degree of association between pairs of items within the scale, offering insight into internal coherence. In parallel, corrected item–total correlations were examined to determine the individual contribution of each item to the overall construct. Lastly, Cronbach’s alpha if item deleted was calculated to evaluate whether the removal of any item would substantially improve the internal consistency of the scale.

### 2.5. Ethical Aspects

This research was conducted in accordance with the ethical standards described in the Declaration of Helsinki [43]. The participants were informed that joining the study was completely voluntary and that they could decide to drop out of the study at any time without penalty. They were also informed that their participation would not result in any personal gain and that all data collected would be kept confidential and anonymous, would be used exclusively for the study, and would not have any adverse effects on them. Their acceptance of participation in this study was documented in a written informed consent. The authorization for this study was obtained from the Research Ethics Committee of the Regional Research Community, Regional Government of Andalusia, on 20 January 2021 (Reference code: PI 036/20).

## 3. Results

### 3.1. Descriptive Analysis

The total study sample consisted of 1197 nursing students residing in Spain. As can be seen in Table 1, of the total sample, 85% were female and 14.6% were male. Also, 0.1% declared themselves to be non-binary, and the rest preferred not to respond to this variable. The age of the sample ranged from 17 to 57 years, with a mean of 22.35 years (SD = 5.783). Regarding the place of residence, most of the participants were from the province of Seville (15.7%), with Guadalajara and Avila being the least represented (0.1%).

Considering the total sample, 78.2% of the nursing students were enrolled in public universities or public university centers, and 28.2% were in their second year of the Bachelor’s degree in Nursing. The mean score for the number of cohabitants in the same household was 3.48 (SD = 0.979). With regard to the perceived level of knowledge during the pandemic, a mean score of 7.33 (SD = 1.654) was obtained. The participants also rated their general health with a mean score of 7.86 (SD = 1.271), even though 73.9% had to isolate themselves at some point due to close contact with infected subjects or infection, and 46.5% reported that they had been diagnosed with COVID-19 at some point. Still, 72.4% of the sample reported not belonging to high-risk groups.

In total, 55.9% of the students stated that their academic contacts of reference had provided them with the necessary self-protection measures to avoid contagion, and 65.2% had received specific training to avoid contagion. Therefore, only 6.5% of the students reported feeling very unsafe or not safe at all during their period of clinical placement.

The overall mean score on the AMICO scale was 4.06 (SD = 1.547), with the observed values ranging between 1 and 9.56 (see Table 1). The Kolmogorov–Smirnov test indicated a significant deviation from normality (*p* < 0.001), suggesting that the distribution of the scores did not conform to a normal curve. As a result, non-parametric statistical methods were applied. In the bivariate analysis, the AMICO total score showed statistically significant differences when compared across variables such as gender, province of residence, household size, perceived general health, COVID-19 diagnosis, risk group status, availability of protective measures during clinical placements, and vaccination history.

Females showed high levels of fear and anxiety on the AMICO scale (4.15) compared to males (3.54) and considering differences in the sample proportions (Table 1). The levels of fear and anxiety were also higher for those students who had never been diagnosed with COVID-19 (4.16), as well as for those who were considered to be at risk (4.37) and those who had not received self-protection measures during their clinical placement (4.12).

### 3.2. Psychometric Analysis

To assess the dimensional structure of the AMICO scale, an exploratory factor analysis (EFA) was conducted. The sampling adequacy was confirmed by the Kaiser–Meyer–Olkin (KMO) measure, which yielded a value of 0.924, while Bartlett’s test of sphericity was significant (*p* < 0.001), indicating the suitability of the data for factor analysis. The analysis resulted in a two-factor structure, corresponding to anxiety and fear, comprising a total of 16 items (see Table 2), and accounting for 88% of the total variance. The internal consistency of the instrument was high, with a Cronbach’s alpha of 0.913. Furthermore, following recent methodological recommendations [44], McDonald’s Omega was also computed, obtaining a value of 0.916.

Subsequently, a CFA was carried out for the construct validity study, which yielded the following values: CFI = 0.910; TLI = 0.910; IFI = 0.900; RMSEA = 0.078; and SRMR = 0.06. To enhance the psychometric analysis of the AMICO scale, three additional tables are included.

Table 3 presents the model fit indices derived from the CFA, along with their interpretation, to assess the adequacy of the factorial structure.

Table 4 presents the inter-item correlation matrix based on Spearman’s rank correlation, providing insight into the internal consistency and coherence among items. The matrix displays the correlations between each pair of items in the AMICO scale, where higher values suggest that the items are likely measuring similar underlying constructs. Overall, the inter-item correlations indicated that most items were positively associated, supporting the assumption that they reflected the related aspects of anxiety and fear concerning COVID-19.

Table 5 displays the corrected item–total correlations and the values of Cronbach’s alpha if each item were deleted, offering further evidence of the individual contribution of each item to the overall reliability of the scale.

Corrected item–total correlations indicate how well each item correlates with the total score of the scale, excluding the item itself. Higher values suggest that the item is a strong indicator of the overall construct being measured. In this study, the corrected item-total correlations were generally high, indicating that each item contributed meaningfully to the internal consistency of the scale.

Cronbach’s alpha measures the internal consistency of a scale, with values above 0.70 typically considered acceptable. The overall Cronbach’s alpha obtained in this study exceeded 0.90, reflecting excellent reliability. Additionally, the values of Cronbach’s alpha if each item were deleted are reported. These values help identify any item that may negatively affect the overall reliability of the scale; however, in this case, all items appeared to support the internal consistency.

**Figure 1:**
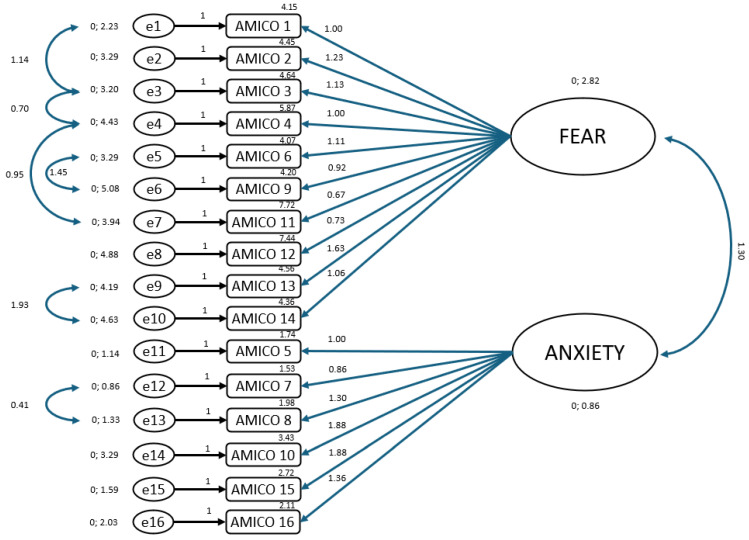
Factorial structure through confirmatory factor analysis.

Figure 1 illustrates the factorial structure of the AMICO scale as confirmed through the CFA. The model supports a two-factor solution comprising “Fear” and “Anxiety”, each associated with their corresponding items. The standardized factor loadings indicated moderate to high relationships between items and their latent constructs, supporting the internal structure of the scale. The correlation between the two latent variables (fear and anxiety) is also shown, suggesting a strong positive association and conceptual coherence between the two dimensions.

## 4. Discussion

The main aim of this study was the psychometric validation of the AMICO scale in nursing students in Spain to specifically create a reliable and valid tool with the aim of detecting levels of fear and anxiety experienced by students as a result of the COVID-19 pandemic. This study provides a comprehensive overview of how various sociodemographic and health-related factors influenced the emotional well-being of nursing students in Spain during the COVID-19 pandemic, using a scale that had previously validated these psychological variables in the general Spanish adult population [29] and also in the UK adult population [45].

The exploratory factor analysis confirmed the two-factor structure of the AMICO scale for nursing students in Spain—namely, anxiety and fear—which together explained 88% of the variance. This finding reinforces the validity of the scale as a reliable instrument for measuring emotional responses related to the COVID-19 pandemic within this healthcare-related student population, in line with previous applications [29,38]. Regarding the reliability analysis, the scale demonstrated an overall Cronbach’s alpha of 0.913, indicating adequate internal consistency. This is consistent with prior validations, such as the AMICO_Pregnant version for pregnant women (*α* = 0.95) [31] and the original version for the adult population (*α* = 0.92) [29]. The high reliability indicated by both Cronbach’s alpha and Omega coefficients further supports the robustness of the scale in this context [46].

The use of this tool in nursing students seeks to detect and prevent possible alterations in emotional well-being that may compromise their academic performance, as well as their mental health. In this sense, it is essential that universities take care of the mental health of students through qualified personnel who can assess possible alterations and address them as soon as possible, as well as through programs aimed at the prevention, detection, and treatment of possible mental health issues [47].

Associations were found between sociodemographic and health factors, including gender, place of residence, and general health status, suggesting that the ability to remain academically motivated and focused during the pandemic was strongly influenced by external constraints and personal experiences [48]. Those in the final stages of their Bachelor’s degree in Nursing, who often faced the additional burden of clinical placements, were particularly vulnerable to reduced engagement, probably due to their increased exposure to harmful healthcare environments during the crisis [49].

In this study, the use of the adapted AMICO scale revealed findings that highlighted significant levels of fear and anxiety experienced by these students, particularly among specific subgroups such as women, those living with more cohabitants, and individuals without a previous diagnosis of COVID-19. This illustrates the complex interaction between personal experience, perceived health risks, and academic pressure during the pandemic [9,12,15].

The bivariate analysis performed for the total AMICO variable showed statistically significant differences in the mean scale score and gender (*p* < 0.001). Previous studies had also found gender differences in relation to anxiety and stress among nursing students [50,51]. In line with this, the study by Graves et al. [52] found that women were more affected by perceived stress than men and were more likely to use coping strategies to manage stress. The high levels of anxiety and fear observed in female students were consistent with global studies suggesting that women generally tend to exhibit higher levels of stress and anxiety during health crises [53].

The levels of anxiety and fear were found to be higher in students who had never been diagnosed with COVD-19 (*p* = 0.028). These data suggest that experiencing the disease provided a sense of security for the students, as opposed to the fear of the unknown.

Another group who reported high levels of anxiety and fear of COVID-19 included those who considered themselves to be in an at-risk group (*p* < 0.001). This classification includes individuals with obesity, COPD or other respiratory diseases, diabetes, older adults, immunocompromised or oncological patients, and pregnant women. These types of conditions have been associated with worse clinical outcomes, most notably death, in COVID-19 patients [54].

The study revealed that the students who had received all three doses of the COVID-19 vaccine reported the highest levels of anxiety and fear on the AMICO scale. While vaccination is typically linked to lower levels of psychological distress, this apparently paradoxical outcome may be explained by a convergence of factors. It is likely that individuals experiencing greater fear of infection were also those most motivated to complete the full vaccination schedule, in line with behavioral theories that associate higher risk perception with increased adherence to preventive health measures [55,56].

Additionally, increased anxiety among the fully vaccinated students may indicate a persistent sense of vulnerability despite immunization, possibly driven by concerns over emerging variants, long-term vaccine efficacy, or the health of vulnerable family members [57]. From a psychological perspective, this response may relate to health anxiety or hypervigilance, where individuals remain preoccupied with health risks even after engaging in preventive measures [58,59].

It is also important to consider the possibility of reverse causality, whereby pre-existing anxiety or health-related concerns may have driven both the decision to complete vaccination and the higher levels of fear reported [58,60]. Moreover, unmeasured psychological factors, such as a predisposition to health anxiety or previous mental health conditions, could act as confounders in this relationship [61]. The influence of contextual variables, such as media exposure, academic demands, and institutional messaging about risk, may further contribute to sustained emotional distress [62]. In this context, our findings suggest that the full vaccination status may serve as an indicator of greater sensitivity to health threats rather than as a marker of emotional reassurance. Further research is needed to explore how individual differences in coping strategies, perceived control, and trust in health information mediate this relationship.

Finally, those nursing students who had not received sufficient self-protection measures during their clinical placements also had the highest levels of anxiety and fear of COVID-19 (*p* < 0.001). All this supports the assumption that perceived vulnerability, whether personal or environmental, played a key role in shaping emotional responses to the pandemic [63]. In the wake of the rapid incursion of the virus into our society, health institutions had to make a colossal effort to meet the increased need for self-protection measures in record time. As a result, there was a shortage of resources and a sense of insecurity at the start of the pandemic. Since the pandemic, the use of self-protection measures has become more widespread in society, thus increasing awareness of these measures and their correct use and fostering social responsibility towards measures against disease transmission [64].

### 4.1. Limitations

Despite presenting important findings regarding the psychometric properties of the AMICO scale in Spanish nursing students and identifying factors associated with their emotional well-being during the COVID-19 pandemic, this study has certain inherent limitations.

First, the use of a non-probabilistic snowball sampling method represents a key limitation [37]. While this approach facilitated the recruitment of a substantial sample of nursing students from most of the provinces across Spain (47 out of 52 provinces), it may have introduced selection bias. As a result, the generalizability of our findings—particularly regarding the prevalence of anxiety and fear and the associations with sociodemographic and health-related factors—should be interpreted with caution when extrapolating the results to the broader population of nursing students in Spain or other contexts.

Second, the cross-sectional design of the study offers only a snapshot in time. Although it provides valuable insights into the emotional state and associated factors among students during the specific period of data collection, it does not allow for an examination of how these variables—such as anxiety, fear, or academic engagement—evolved over time [33]. Given the dynamic nature of the pandemic and the progression through different stages of the nursing program, students’ well-being was likely influenced in a temporal manner. Our data captures a specific moment but do not permit conclusions regarding trends or causal relationships.

Third, one limitation of this study concerns the limited data collected regarding the specific universities or institutions in which the participants were enrolled. Although achieving representativeness across all Spanish nursing schools was not an objective, the study prioritized geographic diversity at the provincial level. However, the absence of institutional data limited the ability to explore potential differences related to factors such as institutional size or variations in academic and clinical training settings. Future research could address this limitation by examining how institutional characteristics may influence nursing students’ psychological responses.

### 4.2. Future Research Perspectives and Practical Implications

Further longitudinal research is essential to gain deeper insight into how the emotional well-being and academic engagement of nursing students evolved throughout the various stages of the COVID-19 pandemic and across the different phases of their nursing training. Beyond examining the temporal progression of anxiety and fear, future studies should also explore the relationship between psychological distress—measured using the AMICO scale validated in this study—and other critical dimensions of well-being affected by the pandemic [32].

The COVID-19 lockdowns and associated restrictions have had profound and enduring effects on quality of life, sleep patterns, and physical activity. For instance, a study conducted in Italy reported a significant decline in perceived quality of life, mental health, and sleep quality, alongside an increase in sedentary behavior several months after the lockdown measures were lifted [65]. Investigating the interplay between anxiety and fear of COVID-19 and these broader aspects of well-being would offer a more comprehensive understanding of the pandemic’s impact on nursing students. This, in turn, could inform the development of more holistic and targeted support strategies [66].

In terms of practical implications, this study highlights the urgent need to implement emotional and academic support systems for nursing students, especially during public health crises. Effectively addressing specific mental health challenges—such as anxiety and fear—among vulnerable subgroups is critical to safeguarding the well-being and future readiness of the nursing workforce [67]. Furthermore, the findings of this study emphasize the importance of providing adequate self-protection measures and clear, transparent communication about health issues to mitigate the psychological impact of crises of this kind on students [68]. These efforts would not only improve students’ outcomes during ongoing challenges but also enhance their professional preparedness in the event of future crises.

Higher education institutions should consider integrating proactive mental health strategies into their emergency preparedness frameworks. This includes establishing accessible psychological counseling services, peer support networks, and mental health literacy programs tailored to health science students [69,70,71]. Training academic staff to identify early signs of distress and fostering a supportive learning environment are also essential [71]. Moreover, incorporating simulation-based learning [72,73] and resilience-building curricula [74] may help students better cope with uncertainty and high-pressure scenarios, ultimately strengthening both academic performance and long-term professional development [75].

## 5. Conclusions

This study highlighted elevated levels of anxiety and fear among nursing students in Spain during the COVID-19 pandemic, particularly among women and those in perceived high-risk situations. The validation of the AMICO scale confirmed its utility for assessing these emotional responses in this population. Notably, students who received all three vaccine doses reported higher anxiety levels, accentuating the need for a more nuanced understanding of the psychological factors influencing health behaviors. These findings point to the urgent need for higher education institutions to implement targeted mental health strategies, especially during public health emergencies. Future longitudinal research should further explore the evolving impact of crises on students’ emotional well-being and examine the role of factors such as quality of life, sleep, and physical activity in mitigating psychological distress.

## Figures and Tables

**Table 1:** Sociodemographic characteristics of the participants related to the study variables.

Quantitative Variables	Mean SD	Range
Age	22.35 5.783	Max = 57 Min = 17
Number of cohabitants	3.48 0.984	Max = 6 Min = 1
General health	7.86 1.271	Max = 10 Min = 1
Total AMICO	4.06 1.546	Max = 9.56 Min = 1
**Qualitative Variables**	**Items**	**Result** **(*N* = 1197)**
Sex	Male Female Non-binary Rather not say	*n* = 175 (14.6%) *n* = 1017 (85%) *n* = 1 (0.1%) *n* = 4 (0.3%)
Academic year	1st year 2nd year 3rd year 4th year	*n* = 304 (25.4%) *n* = 338 (28.2%) *n* = 290 (24.2%) *n* = 265 (22.1%)
COVID-19 diagnosis	No Yes	*n* = 640 (53.5%) *n* = 557 (46.5%)
Isolation	No Yes	*n* = 313 (26.1%) *n* = 884 (73.9%)
High-risk group	No Yes	*n* = 867 (72.4%) *n* = 330 (27.6%)
Self-protection measures	No Yes Other	*n* = 487 (40.7%) *n* = 669 (55.9%) *n* = 41 (3.4%)
Feeling safe during clinical placement	Somewhat safe Fairly safe Very little safe No, not safe at all Yes, completely safe	*n* = 361 (30.2%) *n* = 565 (49.7%) *n* = 56 (4.7%) *n* = 22 (1.8%) *n* = 163 (13.6%)
COVID-19 vaccine	I do not wish to get vaccinated Yes, a single dose Yes, two doses Yes, three doses Not yet vaccinated	*n* = 7 (0.6%) *n* = 42 (3.5%) *n* = 368 (30.7%) *n* = 778 (65%) *n* = 2 (0.2%)

Note. Authors’ self-elaboration. SD: standard deviation; Max: maximum; Min: minimum.

**Table 2:** Exploratory factor analysis of the dimensional structure.

AMICO Scale Items	Component	
	Factor 1	Factor 2
AMICO 1. I am very afraid of COVID-19	0.652	0.403
AMICO 2. I feel uneasiness when thinking about COVID-19	0.607	0.427
AMICO 3. I am very concerned about getting COVID-19	0.695	0.338
AMICO 4. The COVID-19 disease may cause death, and this worries me	0.713	0.166
AMICO 5. My hands sweat when I think about COVID-19	0.132	0.750
AMICO 6. I feel nervous or anxious when watching news or stories about COVID-19 in social networks and other media	0.549	0.512
AMICO 7. I can’t sleep because I am worried about getting COVID-19	0.046	0.833
AMICO 8. My pulse races when I think about getting COVID-19	0.241	0.784
AMICO 9. Contradictory information about coronavirus in social networks and the media makes me feel anxious	0.449	0.440
AMICO 10. I have negative ideas when I hear or read any news related to the disease	0.505	0.540
AMICO 11. I am afraid a relative or friend may get COVID-19	0.702	0.009
AMICO 12. I am worried about how long the pandemic will last	0.669	0.003
AMICO 13. When someone coughs near me, or too close to me, I am afraid of getting infected	0.643	0.321
AMICO 14. I am worried about being close to or assisting a person that has or may have COVID-19	0.628	0.318
AMICO 15. I feel sad or downcast when I think about the disease and the possibility of getting infected	0.408	0.687
AMICO 16. I feel anxious about going out, or considering going out, to fulfil my daily responsibilities (work, family etc.)	0.177	0.701

Note. Authors’ self-elaboration.

**Table 3:** Fit indices and interpretation.

Fit Index	Value	Acceptable Threshold	Interpretation
χ^2^/df	2.5	<3.0	Acceptable
CFI	0.91	≥0.90	Good fit
TLI	0.91	≥0.90	Good fit
IFI	0.90	≥0.90	Acceptable
RMSEA	0.078	≤0.08	Acceptable
SRMR	0.06	≤0.08	Good fit

χ^2^/df (Chi-square/df); CFI (Comparative Fit Index); TLI (Tucker–Lewis Index); IFI (Incremental Fit Index); RMSEA (Root-Mean-Square Error of Approximation); SRMR (Standardized Root-Mean-Square Residual).

**Table 4:** Inter-item correlation matrix (Spearman) of the AMICO scale.

	AMICO_1	AMICO_2	AMICO_3	AMICO_4	AMICO_5	AMICO_6	AMICO_7	AMICO_8	AMICO_9	AMICO_10	AMICO_11	AMICO_12	AMICO_13	AMICO_14	AMICO_15	AMICO_16
AMICO_1	1.0	0.64	0.75	0.56	0.4	0.46	0.36	0.48	0.32	0.45	0.34	0.25	0.51	0.5	0.49	0.38
AMICO_2	0.64	1.0	0.59	0.44	0.4	0.57	0.33	0.43	0.47	0.57	0.28	0.36	0.41	0.43	0.51	0.35
AMICO_3	0.75	0.59	1.0	0.59	0.35	0.45	0.31	0.48	0.31	0.45	0.37	0.31	0.51	0.49	0.47	0.36
AMICO_4	0.56	0.44	0.59	1.0	0.28	0.44	0.22	0.33	0.32	0.38	0.47	0.32	0.39	0.39	0.37	0.25
AMICO_5	0.4	0.4	0.35	0.28	1.0	0.4	0.52	0.56	0.32	0.42	0.16	0.13	0.32	0.31	0.5	0.47
AMICO_6	0.46	0.57	0.45	0.44	0.4	1.0	0.36	0.46	0.6	0.66	0.33	0.37	0.42	0.44	0.53	0.41
AMICO_7	0.36	0.33	0.31	0.22	0.52	0.36	1.0	0.64	0.31	0.42	0.09	0.07	0.3	0.27	0.49	0.51
AMICO_8	0.48	0.43	0.48	0.33	0.56	0.46	0.64	1.0	0.38	0.49	0.2	0.16	0.42	0.42	0.57	0.48
AMICO_9	0.32	0.47	0.31	0.32	0.32	0.6	0.31	0.38	1.0	0.62	0.28	0.37	0.34	0.36	0.42	0.33
AMICO_10	0.45	0.57	0.45	0.38	0.42	0.66	0.42	0.49	0.62	1.0	0.31	0.36	0.43	0.42	0.55	0.42
AMICO_11	0.34	0.28	0.37	0.47	0.16	0.33	0.09	0.2	0.28	0.31	1.0	0.41	0.41	0.35	0.25	0.18
AMICO_12	0.25	0.36	0.31	0.32	0.13	0.37	0.07	0.16	0.37	0.36	0.41	1.0	0.34	0.3	0.28	0.16
AMICO_13	0.51	0.41	0.51	0.39	0.32	0.42	0.3	0.42	0.34	0.43	0.41	0.34	1.0	0.67	0.48	0.38
AMICO_14	0.5	0.43	0.49	0.39	0.31	0.44	0.27	0.42	0.36	0.42	0.35	0.3	0.67	1.0	0.52	0.36
AMICO_15	0.49	0.51	0.47	0.37	0.5	0.53	0.49	0.57	0.42	0.55	0.25	0.28	0.48	0.52	1.0	0.6
AMICO_16	0.38	0.35	0.36	0.25	0.47	0.41	0.51	0.48	0.33	0.42	0.18	0.16	0.38	0.36	0.6	1.0

**Table 5:** Corrected item–total correlations and Cronbach’s alpha if item deleted.

Item	Item–Total Correlation	Cronbach’s Alpha If Item Deleted
AMICO_1	0.745	0.904
AMICO_2	0.741	0.904
AMICO_3	0.742	0.904
AMICO_4	0.657	0.908
AMICO_5	0.563	0.91
AMICO_6	0.755	0.904
AMICO_7	0.547	0.911
AMICO_8	0.667	0.908
AMICO_9	0.644	0.909
AMICO_10	0.737	0.904
AMICO_11	0.554	0.911
AMICO_12	0.537	0.912
AMICO_13	0.703	0.906
AMICO_14	0.692	0.907
AMICO_15	0.744	0.904
AMICO_16	0.578	0.91
Overall		0.913

## Data Availability

Data available under reasonable request to the corresponding author.

## Abbreviations

The following abbreviations are used in this manuscript:

AMICO	Anxiety and Fear to COVID-19 Scale
CAS	Coronavirus Anxiety Scale
CSS	COVID-19 Stress Scale
FCV-19S	Fear of COVID-19 Scale
SPSS	Statistical Package for the Social Sciences
KMO	Kaiser–Meyer–Olkin test
EFA	Exploratory Factor Analysis
SD	Standard Deviation
IFI	Incremental Fit Index
TLI	Tucker–Lewis Index
CFI	Comparative Fit Index
RMSEA	Root-Mean-Square Error of Approximation
STROBE	Strengthening the Reporting of Observational Studies in Epidemiology
COPD	Chronic Obstructive Pulmonary Disease
UK	United Kingdom
eHEALS	eHealth Literacy Scale
